# Crystal structure of ultra-humanized anti-pTau Fab reveals how germline substitutions humanize CDRs without loss of binding’

**DOI:** 10.1038/s41598-022-12838-6

**Published:** 2022-05-24

**Authors:** Alette R. Brinth, Kristine Svenson, Lidia Mosyak, Orla Cunningham, Timothy Hickling, Matthew Lambert

**Affiliations:** 1 BioMedicine Design, Pfizer Worldwide R&D, Dublin, D22 V8F8 Ireland; 2 BioMedicine Design, Pfizer Worldwide R&D, Cambridge, MA 02139 USA; 3 BioMedicine Design, Pfizer Worldwide R&D, Andover, MA 01810 USA; 4 Ultrahuman Ltd. Kreston Reeves LLP Innovation Hs, Ramsgate Rd, Sandwich, CT13 9FF UK

**Keywords:** Drug discovery, Biologics, X-ray crystallography

## Abstract

Administration of therapeutic antibodies can elicit adverse immune responses in patients through the generation of anti-drug antibodies that, in turn, reduce the efficacy of the therapeutic. Removal of foreign amino acid content by humanization can lower the immunogenic risk of the therapeutic mAb. We previously developed the ultra-humanization technology “Augmented Binary Substitution” (ABS) which enables single-step CDR germlining of antibodies. The application of ABS to a chicken anti-pTau antibody generated an ultra-humanized variant, anti-pTau C21-ABS, with increased human amino acid content in the CDRs and reduced in-silico predicted immunogenicity risk. Here, we report the high-resolution crystal structure of anti-pTau C21-ABS Fab in complex with the pTau peptide (7KQK). This study examines how ultra-humanization, via CDR germlining, is facilitated while maintaining near-identical antigen affinity (within 1.6-fold). The co-complex structure reveals that the ABS molecule targets the same antigenic epitope, accommodated by structurally-similar changes in the paratope. These findings confirm that ABS enables the germlining of amino acids within CDRs by exploiting CDR plasticity, to reduce non-human amino acid CDR content, with few alterations to the overall mechanism of binding.

## Introduction

Monoclonal antibodies (mAbs) are a well-established and growing category of therapeutics, representing over half of first-time biopharmaceutical approvals in the period January 2015 to July 2018^1,2^. However, their administration in the clinic has been complicated by the fact that therapeutic mAbs can elicit adverse immune responses in patients by generating anti-drug antibodies (ADAs)^3,4^. ADAs can affect the efficacy of a therapeutic protein in vivo through a reduction in serum drug concentrations or drug neutralization. In more serious cases, ADAs can even induce hypersensitivity responses such as anaphylaxis^5–8^.

There are a range of both extrinsic and intrinsic factors that may contribute to the immunogenic potential of a therapeutic mAb^9,10^. Extrinsic factors such as aggregates and adjuvant-like contaminants can be addressed by improvements in manufacturing and formulation of the mAb^11^. Factors intrinsic to the mAb are more difficult to address and may require extensive engineering efforts to remove potential T-cell epitopes.

One method for lowering ADA generation is to reduce the presence of non-human amino-acid content in the therapeutic mAb^12^. Antibody humanization is therefore an essential engineering step in the pre-clinical development phase. In the classical form of antibody humanization, non-human complementarity-determining regions (CDRs) are grafted onto human V-gene sequences^13^. CDR-grafting frequently requires the re-introduction of “parental” residues, at key positions, into framework-regions (back-mutations) to re-establish CDR orientation and antigen affinity. Nevertheless, humanized mAbs do present with lower immunogenicity risk in patients in comparison to chimeric (non-human V-genes fused to fully human constant regions) mAbs^12^.

However, antibodies discovered from human, naïve repertoires, such as Adalimumab and Golimumab, can demonstrate significant levels of immunogenicity^14,15^. One reason for this is attributed to non-germline amino acids located within the CDRs of the mAb^11^. ADAs induced by humanized mAbs are predominantly anti-idiotypic antibodies, targeted at the CDRs^10^.

CDRs have previously been regarded as non-malleable due to the anticipated risk of antigen affinity loss^11,16,17^. Recent studies have now proven that it is possible to mutate amino acids within the CDRs of mAbs without reducing affinity or potency^18^.

We previously developed the humanization technology “Augmented Binary Substitution” (ABS)^19^. The ABS process increases CDR germline content while maintaining epitope specificity and antigen affinity. The method is described in detail in Townsend et al.^19^. Briefly, binary substitution cassettes from host species are inserted into human frameworks to create combinatorial libraries in which only the parental or human germline residue are encoded at each position. The CDR-H3 cassette is augmented with 1 ± 1 random substitution per clone. The combinatorial library is screened for clones that have parental antigen-binding affinity^19^. Using ABS, the maximum number of CDR residues are germlined without compromising antigen binding. The “humanness” of the mAb is increased through restoration of 100% germline frameworks in combination with a significant return to germline within CDRs, thus reducing the overall immunogenic risk of the ultra-humanized mAb.

Using ABS, we ultra-humanized a chicken anti-pTau antibody, a mAb recognising phosphorylated tau (a constituent of the non-fibrillar tangles in Alzheimer’s patients)^20^. This resulted in the ultra-humanized variant anti-pTau ABS-C21, which has an increased CDR germline content of 65% compared to 41% in the parental antibody^19^. Although the amino acid content of the CDRs changed, we were able to confirm that the binding affinity remained within 1.6-fold of one another, as measured by SPR (0.41 nM and 0.25 nM respectively)^19^.

Through removal of any extraneous non-germline, amino acid content, ultra-humanization of anti-pTau defined the minimal functional paratope. However, up until now, the process by which the paratope was altered to maintain affinity/specificity remains unclear. We hypothesized that CDR germlining could be achieved through the establishment of a new network of antigen contacts mediated by the sidechains of newly introduced human amino acids or that tolerated amino acid changes indicated positions not forming contacts with the pTau peptide.

Here, we report the crystal structure of anti-pTau ABS-C21 Fab in complex with pTau peptide (Protein Data Bank (PDB) ID 7KQK) to empirically establish how the architecture of the paratope/epitope binding interface is modified to accommodate the ABS CDR germlining process. The co-complex structure reveals an unaltered epitope and the changes in the paratope facilitating binding. We show that ABS enables CDR germlining through the exploitation of CDR plasticity, thus minimizing non-human amino acid CDR content while maintaining the overall mechanism of binding.

## Results

### Crystallisation of the anti-pTau C21-ABS Fab in complex with pTau reveals a distinct paratope

To investigate how ultra-humanisation of anti-pTau altered the paratope and epitope interface, we generated purified anti-pTau C21-ABS Fab (Figs. S1, S2A) and crystallized it in complex with the pTau peptide (Fig. S2B). As previously reported by Townsend et al.^19^, humanization of Anti-pTau to C21 maintained affinities within 1.6-fold (K_DWT_ = 0.41 nM, KD_C21_ = 0.25 nM).

The sequence of the pTau peptide (^224^KKVAVVR(pT_231_)PPK(pS_235_)PSSAKC^241^) was the same as that previously crystallized in complex with the anti-pTau WT Fab (PDB ID 4GLR)^20^. The structure of the anti-pTau C21-ABS Fab in complex with the pTau peptide was solved to a resolution of 2.6 Å (Table S1). Two complexes are present in the asymmetric unit, displaying identical binding modes. Analysis is presented of chains H, L and P.

**Figure 1:**
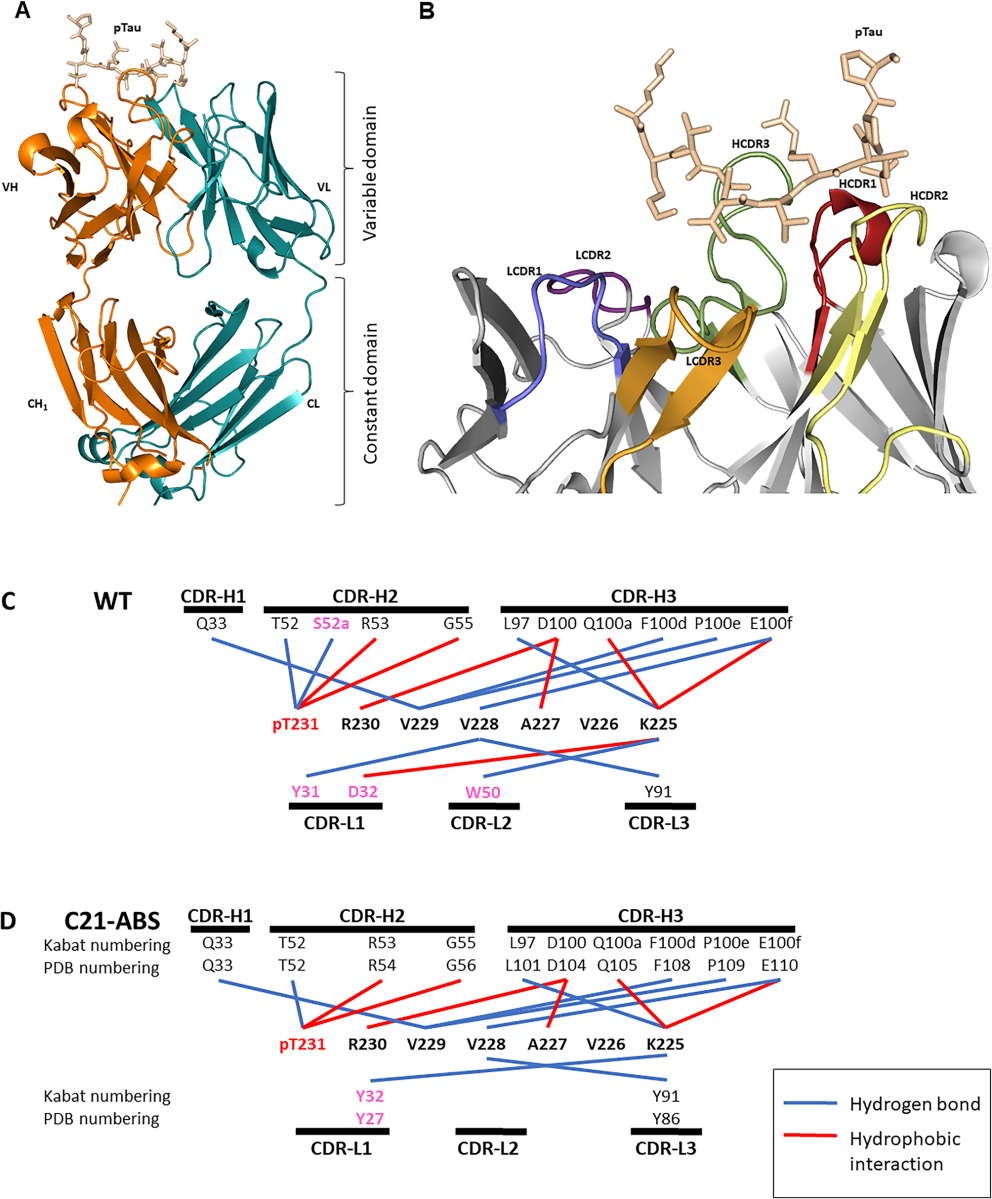
Structure of Anti-pTau-C21 with Bound pTau Peptide. (**A**) Architecture of the Anti-pTau C21 Fab fragment comprising the V_H_ (orange: V_H_ + C_H_1) and V_L_ (cyan: V_L_ + C_L_) chains. pTau is depicted in wheat. (**B**) Detailed view of the variable domain showing HCDR1 (red), HCDR2 (yellow), HCDR3 (green), LCDR1 (blue), LCDR2 (purple) and LCDR3 (orange). (**C**) Schematic representation of all contacts between pTau and the anti-pTau WT Fab structure and (**D**) the pTau and the anti-pTau C21-ABS structure. Variations in amino acid sequence have been coloured magenta.

**Figure 2:**
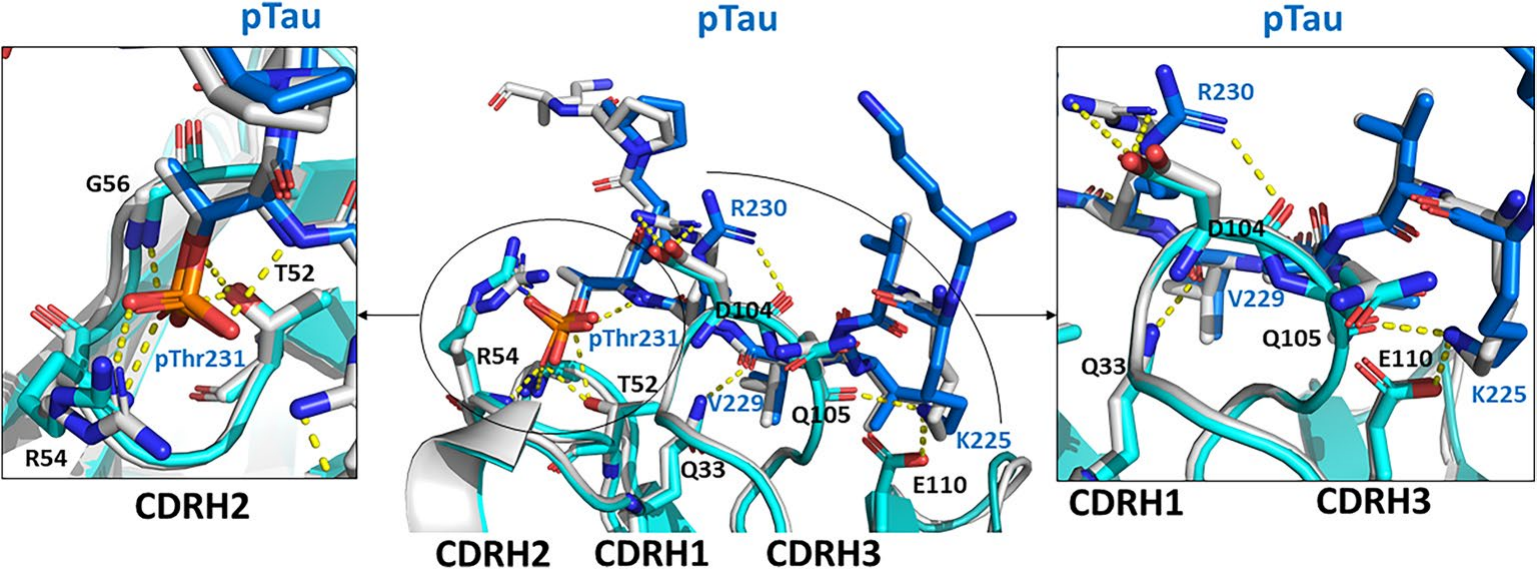
Overlay of WT and ABS-C21 Anti-pTau with bound pTau peptide. Alignment of anti-pTau WT (cyan) and ABS-C21 Fab (grey) in complex with the pTau peptide (blue). Non-interface portions of the Fabs are shown in cartoon representation. Both the pTau peptide and any Fab interface residues are shown in stick configuration. (Left) Key interactions are displayed between the pTau phosphate group and T52, R53 and G55 in HCDR2. (Right) Key interactions are displayed between pTau_V229_ and HCDR1_Q33_, pTau_R230_ and HCDR3_D104_ and pTau_K225_ with HCDR3_Q105_ as well as HCDR3_E110_.

In the anti-pTau C21-ABS Fab-pTau co-crystal structure (PDB ID 7KQK), 10 amino acids from the pTau peptide are visible (^224^KKVAVVR(pT_231_)PP^233^) (Fig. 1A,B). The remaining 8 (^234^ K(pS_235_)PSSAKC^241^) are disordered. The positioning of the pTau peptide is identical in the anti-pTau WT Fab and ABS-C21 co-crystal structures (Fig. S3). The pTau peptide is positioned above the heavy chain CDRs (Fig. 1A,B) with two sharp turns at Val228_pTau_ and pThr231_pTau_ (Fig. 2). Comparative analysis of the two crystal structures (WT and ABS-C21) shows that the pTau epitope remains unchanged, comprising ^225^KVAVVR(pT) ^231^ (Fig. 1C,D). The whole Fabs do not superimpose well due to conformational flexibility of the Fab’s elbow angles, which is not unexpected. Accordingly, superimposing the Fv fragments only (consisting of the variable domains of the light and heavy chains) we obtained r.m.s.d. values of between 0.12 and 0.3 Å, for the heavy and light chains respectively, indicating the high degree of similarity between the two crystal copies of Fab + pTau.

In both structures, the Fab fragment recognizes six amino acids N-terminal to the Thr_231_ phosphorylation site via CDR-H3, with CDR-H2 dominating the phospho-epitope through interaction with pThr231_pTau_ itself. CDR-H1 and the light chain CDRs provide secondary support (Fig. 1C,D). The anti-pTau paratope structure remains highly similar after the ultra-humanisation process with very few individual interactions disrupted or altered. Overall, there is a removal of 1 × VH (S52a_CDRH2_ (PDB # = 53)) and 2 × VL (Y31_CDRL1_ (PDB # = 26) and W50_CDRL2_ (PDB # = 50)) interactions in the paratope after ultra-humanization of anti-pTau WT to anti-pTau C21-ABS (Fig. 1C,D). Additionally, there is a change in the type of molecular interaction between LCDR1 and HCDR2 as a result of the amino acid substitutions arising from germlining. The mutation D32Y_CDRL1_ (PDB # = 27) results in a change from a salt-bridge in wild-type to hydrophobic interaction with K225_pTau_ in C21.

In LCDR1, 4 out of 6 amino acid residues have been humanized, with the germline residue being positively selected (Table S2). Two of these, Y31_WT_ and D32_WT_, were identified as contact points in the WT co-complex structure, forming a hydrophobic interaction with V228_pTau_ and salt bridge interaction with K225_pTau_, respectively (Fig. 1C). In the ABS-C21 variant, these have been mutated to S31_C21_ (PDB # = 26) and Y32_C21_ (PDB # = 27) (Fig. 1D). This suggests that they are support residues rather than key contact points in the anti-pTau paratope. V228_pTau_ interacts with two other anti-pTau residues (E100f_CDRH3_ (PDB # = 110) and Y91_CDRL3_ (PDB # = 86)), thereby facilitating the Y31S_CDRL1_ (PDB # = 26) mutation. Interestingly, the D32Y_CDRL1_ (PDB # = 27) mutation introduces a new hydrophobic interaction with K225_pTau_. In this way, the positioning of the pTau peptide is maintained. The loss of negative charge that occurs through the D32Y_LCDR1_ (PDB # = 27) mutation is not significant as the overall negative charge of this surface area is maintained by E100f_HCDR3_ (PDB # = 110), which in turn maintains a salt-bridge interaction with K225_pTau_ (Figs. 2, S4).

2 out of 4 amino acid residues have been humanized in LCDR2 (Table S2). One of these, W50_WT_, was identified as a contact point in the WT paratope, forming a hydrophobic interaction with K225_pTau_. The W50G (PDB # = 50) mutation disrupts this interaction by replacement of the bulky Trp sidechain with the much smaller Gly sidechain. However, K225_pTau_ is positionally maintained through three interactions with HCDR3 (L97 (PDB # = 101), Q100a (PDB # = 105) and E100f. (PDB # = 110)) and one with LCDR1 (Y32 (PDB # = 27)), thereby facilitating the mutation of W50G (PDB # = 50) without significant loss of antigen binding (K_D_ within 1.6-fold). Two out of 9 chicken residues are mutated to germline amino acids in LCDR3. Importantly, Y91_WT_ (PDB # = 86) is not mutated, forming a hydrophobic interaction with V228_pTau_ which remains undisrupted in the C21-ABS Fab-peptide co-complex structure (Fig. 2). Interestingly, Y91 (PDB # = 86) was the only LCDR residue that was 100% maintained in our previous analysis of selection output clones from the ultra-humanisation process^19^. The preservation of this residue would suggest its intimate involvement in ligand binding, and this is confirmed with our structural analysis.

Overall, HCDR1 plays a support role in the anti-pTau paratope. However, neither of the two chicken residues present in this CDR are mutated to germline through the ultra-humanization process (Table S2). The HCDR1 chicken residue Q33 (PDB # = 33) is a key contact residue forming a sidechain hydrogen bond with V229_pTau_ which is preserved in the anti-pTau C21-ABS structure (Fig. 2). In agreement with this, we previously observed Q33 (PDB # = 33) to be a “strongly maintained” residue after library selection, with 100% of output clones favoring this residue^19^.

In CDRH2, 6 out of 11 amino acid residues have been humanized (Table S2). Of these, only S52a (PDB # = 53) comprises a contact residue in the WT structure, forming a hydrogen bond with pThr_231_ (Fig. 1C). This has been mutated to Gly in the ABS-C21 structure, with the hydrogen bond being disrupted as a result of the Ser sidechain being replaced with the much smaller Gly amino acid sidechain. This has limited effect on antigen affinity, however, as 3 other hydrogen bond interactions in total are formed between CDRH2 and pThr231 through T52_C21_ (PDB # = 52), R53_C21_ (PDB # = 54) and G55_C21_ (PDB # = 56) (Figs. 1D, 2). Interestingly, the S52aG (PDB # = 53) mutation is not favored in our original sequence analysis of ABS selection output clones, showing > 90% retention across the library. Although unfavored, the bonds supplied by the neighboring amino acid residues T52_C21_ (PDB # = 52), R53_C21_ (PDB # = 54) and G55_C21_ (PDB # = 56)allow for this mutation (Figs. 1D, 2).

No residues in HCDR3 are mutated and the overall loop structure remains identical to the WT structure. E100f_WT_ (PDB # = 110) maintains a critical salt bridge interaction with K225_pTau_ and hydrogen bond with V228_pTau_, positioning the pTau peptide with a sharp turn at V228_pTau_ (Fig. S4). Taken together, these observations confirm our previous hypothesis that only functionally required CDR content is retained after mAb ultra-humanization.

### Comparison of the anti-pTau WT and C21-ABS mAb surface charge distribution reveals the importance of electrostatic interactions in the phospho-epitope

**Figure 3:**
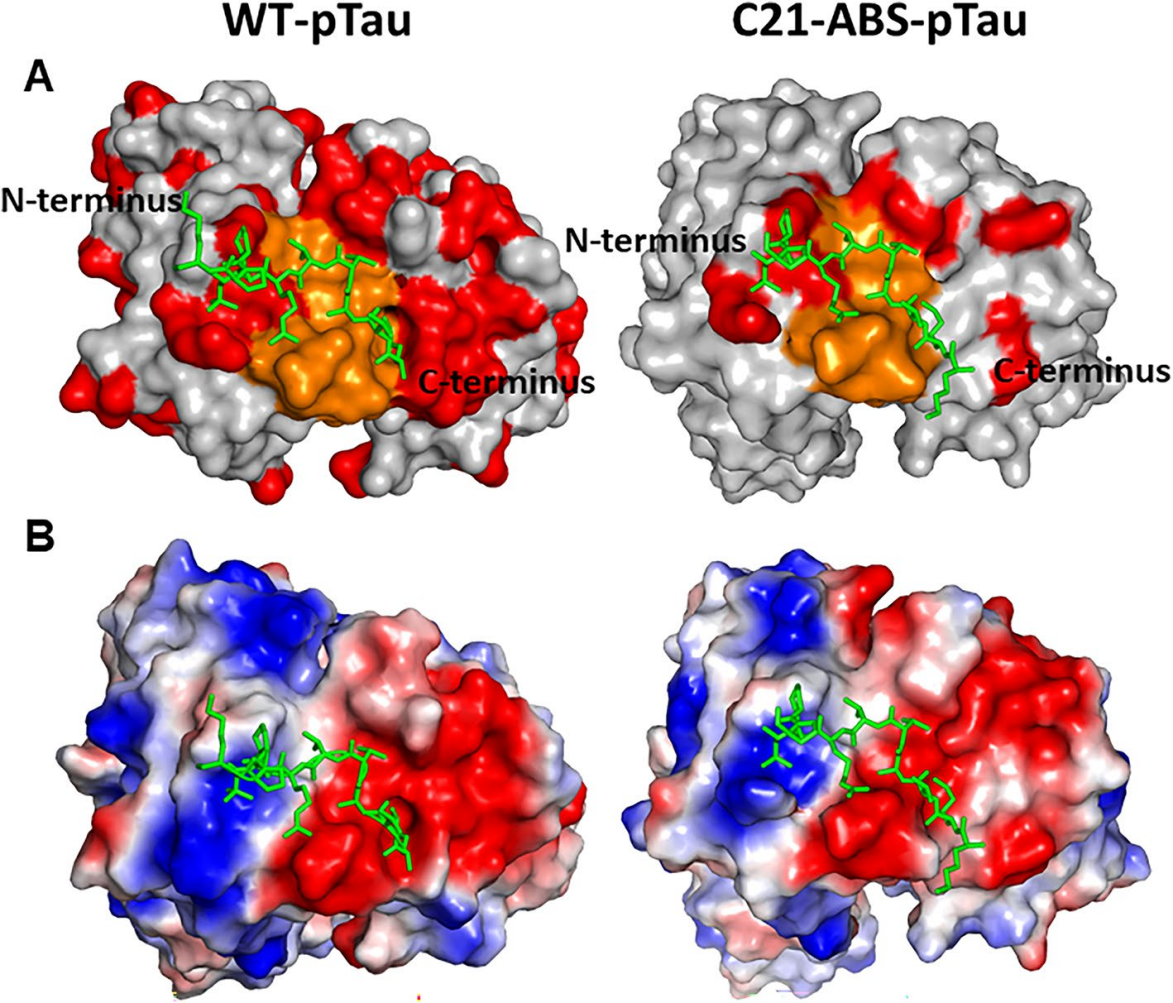
Surface representation of the Anti-pTau WT and ABS-C21 paratopes. (**A**) CDR germlining illustration of WT and ABS-C21 anti-pTau displayed in surface representation, showing non-germline amino acid content. The pTau peptide is displayed in stick configuration. Non-germline, germline and CDR-H3 amino acid residues are shown in red, grey and orange, respectively. (**B**) Surface representation of anti-pTau WT and ABS-C21 showing electrostatic charge distribution. Negatively- and positively charged residues are displayed in red and blue, respectively. Colour shade represents the relative charge distribution across the surface of the variable domain.

To determine the percentage reduction in the presence of non-germline amino acid solvent accessible surface area (ngSASA) on the Fab fragment, jsPISA (Protein Interfaces, Surfaces & Assemblies) analysis was carried out using the CCP4 software suite^21^. In comparison to the parental molecule, the ngSASA was reduced from 2599.9 Å^2^ (WT) to 1882.5 Å^2^ (C21-ABS). This is a difference of 27.5%, representing a significant reduction in the presence of non-germline amino acids on the surface of the anti-pTau C21 Fab fragment (Fig. 3A). Despite the significant reduction in ngSASA, key amino acid residues in the paratope remain unchanged. Very few molecular interactions are altered in the paratope following the ultra-humanization process (Fig. 1C,D). Importantly, the overall electrostatic surface-charge distribution of anti-pTau WT and C21-ABS also remains similar despite the reduction in ngSASA (Fig. 3B). This clearly demonstrates the importance of electrostatic interactions in pTau recognition by the anti-pTau Fab fragment. In total, 4 amino acids (R53_CDRH2_, D100_CDRH3_, E100f_CDRH3_ and D32_LCDR1_) in the anti-pTau WT paratope contribute to electrostatic interactions (Fig. 1C).

As observed in the anti-pTau WT structure, the pTau phosphorylation site pThr_231_ is exclusively recognized by CDR-H2, facilitated by the formation of a positively-charged pocket to accommodate the phosphate group^20^. The positive charge in this pocket is contributed by R53_C21_ (PDB # = 54) (Fig. S4). In our analysis of ABS selection output clones, R53_C21_ (PDB # = 54) was 100% maintained across all clones, indicating that this residue is crucial to antigen specificity. This is clearly explained through the observation in both WT and ABS-C21 crystal structures that this residue makes up the positive charge in the CDRH2 pocket.

In CDRH3, D100_C21_ (PDB # = 104) and E100f_C21_ (PDB # = 110) form negatively-charged patches positioned by the N-terminus of the pTau peptide (Fig. S4). D100_C21_ (PDB # = 104) and E100f_C21_ (PDB # = 110) form salt-bridge interactions with R230_pTau_ and K225_pTau_, respectively (Fig. 1D). These residues are both maintained throughout the ultra-humanization process, illustrating their importance in maintaining antigen affinity.

D32Y (PDB # = 27) is the only amino acid mutation introduced by the ultra-humanization process that disrupts an electrostatic interaction. This mutation disrupts a salt-bridge interaction with K225_pTau_. This agrees with our previous analysis of the ABS selection output clones in which D32_WT_ had a ~ 50% retention rate, indicating that this residue is not crucial for antigen binding. In the anti-pTau ABS-C21 variant, this residue has not been retained. The K225_pTau_ interaction with E100f_C21_ (PDB # = 110) appears sufficient to maintain the positioning of the pTau peptide through a salt-bridge interaction with K225_pTau_ (Fig. 1D). Furthermore, this residue maintains the negatively-charged surface of the anti-pTau Fab required to accommodate K225_pTau_ (Fig. S4).

### The presence of in-silico predicted T-cell epitopes are reduced in anti-pTau C21 by ABS

When analyzing the immunogenic potential of a mAb, one of the first steps is to use in-silico tools to predict the presence of possible T-cell epitopes. For this application, software algorithms and machine-learning methods have been employed to carry out systematic assessments of MHC class II peptide-binding domains within proteins^22^.

**Figure 4:**
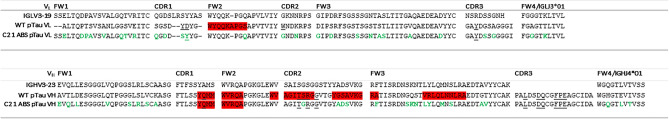
In-silico T-cell epitope predictions for Anti-pTau WT and ABS-C21. Sequence alignment of anti-pTau WT and ABS-C21 with germline VL (IGLV3-19) and VH (IGHV3-23) V-regions. Predicted T-cell epitopes were defined as 9mer peptides that belong to the top 1% of binders for any given allele or the top 5% of binders for at least 4 different alleles amongst 8 DRB1 superfamily alleles. Red indicates in silico predicted T-cell epitopes. Non-human residues that have been changed to germline are bolded and colored green. Residues that are identified as interacting with pTau are underlined.

In-silico predictions for T-cell epitopes within the V-genes of anti-pTau WT and C21-ABS were performed using EpiMatrix (Epivax software, RI)^19^ (Fig. 4). Predicted T-cell epitopes were defined as peptides that belong to the top 1% of binders for any given allele or the top 5% of binders for at least 4 different alleles amongst 8 DRB1 superfamily alleles.

Following ultra-humanization of anti-pTau, the total number of predicted T-cell epitopes was reduced from 5 in anti-pTau WT (1 VL and 4 VH epitopes) to 1 (0 VL and 1 VH epitope) in anti-pTau C21-ABS. The remaining predicted T-cell epitope is present in HCDR1 (Fig. 4). In HCDR1, Q_33_ and M_35_ are the only two chicken residues that were alternatively mutated between WT and germline amino acids in the ABS library (Table S2). Both residues were > 90% maintained in our analysis of ABS selection output clones^19^, indicating that they are important residues for antigen binding. The structure of anti-pTau C21-ABS in complex with the pTau peptide confirms that Q_33_ is a key contact residue, forming a hydrogen bond with V229_pTau_. Due to the importance of this interaction, it was therefore not possible to mutate this amino acid without disruption of antigen binding (Fig. 1C,D).

These in-silico predictions indicate a lowered risk of immunogenicity in the ultra-humanized molecule anti-pTau C21-ABS. It is therefore likely that this ultra-humanized mAb will elicit a weaker immunogenic response in vivo, thus potentially avoiding complications of ADA and reduced half-life.

## Discussion

Antibody humanization is an important step in therapeutic mAb development. The presence of T-cell epitopes can, among other factors, influence the immunogenic potential of a mAb, thereby enhancing the production of ADA^5^. In order to mitigate immunogenicity risks, it is therefore important to minimise the presence of foreign amino acid sequence in antibodies^22^. ABS enables the development of ultra-humanized mAbs with increased CDR germline content and desirable drug-like properties. Until now, the extent to which CDR germlining effects paratope/epitope interactions was unknown.

In this study, we report the crystal structure of the ultra-humanized anti-pTau C21-ABS Fab in complex with pTau peptide. This structure provides, for the first time, a molecular insight into how ultra-humanization, by ABS, is achieved. We see that the paratope/epitope interface is largely unaltered, but where it is, the mAb can compensate elsewhere in the paratope to maintain a functional interaction, thus maintaining affinity (within 1.6-fold, as measured by SPR).

Detailed comparison of the anti-pTau WT and ABS-C21 Fab co-crystal structures reveals a largely unaltered anti-pTau paratope fold following CDR germlining. The HCDR2 surface maintains the same bowl-like pocket observed in the anti-pTau WT interaction with pTau. This HCDR2 fold accommodates the phosphate group of pThr_231_, thereby facilitating the positioning of the pTau peptide through a network of hydrogen bond interactions. As observed in the WT structure, the long HCDR3 loop provides the majority of the anti-pTau contacts. In this way, the peptide is positioned identically above the heavy chain CDRs in the pre- and post-ABS co-crystal structures.

These observations show that CDR germlining of the anti-pTau mAb is enabled through a passive process, in which only functionally required CDR content is retained. The introduction of germline amino acid mutations has not unduly altered the structure of the folded paratope, nor introduced a new network of amino acid sidechain interactions to accommodate any loss of interactions arising from the germlining process. These findings are in agreement with previously published observations that only a small number of CDR residues constitute the minimum binding region of the antibody interacting with the antigen, thereby bestowing a certain level of “plasticity” upon the CDRs outside of these regions^23^. This CDR plasticity is exploited by the ABS process to germline residues that are not directly involved in antigen binding.

Residues involved in the anti-pTau WT paratope that have been mutated are located solely in the light chain, comprising Y31S_LCDR1_, D32Y_LCDR1_ and W50G_LCDR2_. These residues interact with V228_pTau_ and K225_pTau_, which in turn are well-anchored through hydrogen-bond and salt-bridge interactions with multiple residues in HCDR3. Thus, the germlining of Y31, D32 and W50 is facilitated in the anti-pTau light chain without affecting antigen binding/affinity due to their nature as “support” interactions as opposed to key paratope contacts.

It is generally accepted that non-human amino acid residues at the surface of an antibody carry the immunogenic potential of a mAb^24^. To determine the percentage change in non-germline amino acids at the surface of anti-pTau between the WT and ABS-C21 variant, PISA analysis was performed. The reduction in ngSASA of 27.5% from 2599.9 to 1882.5 Å^2^ demonstrates a significantly reduced presence of non-germline amino acids on the surface of the anti-pTau C21 Fab fragment, indicating a putatively lowered immunogenicity risk in the ultra-humanized variant. This significant reduction in ngSASA is facilitated without altering key amino acid residues in the paratope. Significantly, analysis of the electrostatic charge distribution across the Fab surface reveals that the positively-charged pocket formed by CDR-H2 to accommodate pThr_231_ remains. Similarly, the negatively-charged area formed by CDR-H3 is also maintained. Thus, despite a significant increase in germline amino acids at the surface of the Fab, key amino acid residues contributing to electrostatic surface charge distribution are maintained. This corroborates the hypothesis that ABS exploits CDR plasticity to germline residues that are not involved in antibody-antigen interactions. This will inform future CDR germlining efforts, affording an insight into the method by which the altered CDRs facilitate folding of the functional paratope.

The likelihood that this ultra-humanized mAb is less immunogenic than its precedent is further strengthened by the fact that there is a significant reduction in the number of in-silico, predicted T-cell epitopes present in both heavy and light chains. It is interesting to postulate that a decrease in immunogenicity may well be due to a reduction in the polyspecificity of the molecule. It is indeed established in the field, that positive-charge patches can lead to an overall low-level non-specific binding phenotype. However, as has been shown, the overall electrostatic charge distribution of the molecule remains constant and so the WT and ABS mAbs will likely have identical specificity profiles.

In summary, this study has provided an insight into the molecular mechanisms by which ABS enables the ultra-humanization of mAb CDR regions while maintaining antigen affinity. Key residues involved in the paratope remain unchanged, while non-essential residues in the CDR are germlined, thereby increasing the “humanness” of the antibody and simultaneously decreasing immunogenicity risk of the mAb. Despite changes in the residues interacting with pTau, the overall charge distribution across the surface of the Fab is maintained and the lost interactions of the WT molecule with pTau are substituted in the ABS molecule thus maintaining the *status quo*.

## Materials and methods

### IgG purification and Fab preparation

Anti-pTau WT and C21-ABS IgGs were expressed by transient transfection of HEK cells using an Expifectamine 293 Transfection Kit (Thermo Scientific). Antibody was purified from conditioned medium using an ÄKTA Pure (GE Healthcare). Conditioned medium was loaded onto a 1-ml HiTrap mAb SelectSuRE column (GE Healthcare) pre-equilibrated in 20 mM Tris, pH 7.0. The column was washed with five column volumes 20 mM Tris, pH 7.0. Finally, IgG was eluted using 0.1 M glycine, pH 2.7. Peak fractions were concentrated and applied to a HiLoad 26/600 Superdex 200 pg column (GE Healthcare), pre-equilibrated in 20 mM Tris, pH 7.0, 100 mM NaCl. Fab fragments were prepared and purified using a Pierce Fab preparation kit according to manufacturer’s instructions (Thermo Scientific). Purified Fab fragments were concentrated to 14.9 mg/ml for crystallisation trials in a final buffer of 20 mM Tris–HCl, 100 mM NaCl (pH 7.0).

### Crystallisation

Purified anti-pTau C21-ABS Fab was incubated with 10 mM phosphopeptide (^224^KKVAVVR(pT^231^)PPK(pS^235^)PSSAKC^241^) for 1 h at 4 °C immediately prior to crystallisation trials. Phosphopeptide was purchased from BioSciences. The Fab/peptide molar ratio was 1:1.2. Initial crystallisation screens were carried out at 4 °C and 20 °C with a Mosquito robot (TTP Labtech) using high throughput crystallisation screening kits (Hampton Research) at a 1:1 ratio of Fab/peptide mixture and reservoir solution. Crystals of anti-pTau ABS-C21 in complex with pTau grew in 29 days in a reservoir containing 20% (w/v) PEG 3350 and 150 mM DL-malic acid, pH 7.0. Manual optimizations were performed at 20 °C by hanging-drop vapour-diffusion. Crystals were harvested using a cryo-solution comprising 20% (w/v) PEG3350, 150 mM DL-malic acid and 20% ethylene glycol, pH 7.0.

### Data collection, structure solution and refinement

X-ray diffraction data were collected at ALS IMCA-CAT beamline 17-ID. The data were processed with autoPROC. The structure of the anti-pTau C21-ABS Fab-pTau complex was solved by molecular replacement using Phaser with anti-pTau WT (PDB ID 4GLR) as the search model. Crystallographic refinement, alternating with model rebuilding and refitting into 2*F*_*c*_–*F*_*c*_ and *F*_*o*_–*F*_*c*_ electron density maps with Coot, was carried out using autoBUSTER. All structural figures were prepared using PyMOL (DeLano Scientific LLC). Crystal data and refinement statistics are summarized in supplemental Table S1.

### jsPISA (protein, surfaces, and interfaces) analysis

The percentage ngSASA (non-germline Solvent Accessible Surface Area) of anti-pTau C21-ABS was calculated using jsPISA in the Collaborative Computational Project No. 4 software suite (CCP4 (http://www.ccp4.ac.uk/)). The ngSASA is calculated as the surface area of non-germline amino acids relative to the surface area of germline amino acids, and is measured in Å^2^.

## Supplementary Information

Supplementary Information 1.

Supplementary Information 2.

Supplementary Information 3.

Supplementary Information 4.

Supplementary Information 5.

## Data Availability

The datasets used and/or analysed during the current study available from the corresponding author on reasonable request.
