# Supplementary material for: Crystal structure of ultra-humanized anti-pTau Fab reveals how germline substitutions humanize CDRs without loss of binding’
Source: Sci Rep. 2022 May 24;12:8699. doi: 10.1038/s41598-022-12838-6 (PMC9130293; doi:10.1038/s41598-022-12838-6)
Supplement: Supplementary file 1 — Supplementary Information 1. [file 41598_2022_12838_MOESM1_ESM.docx]

# Supplemental Material

**Figure S1. Purification of anti-pTau ABS-C21**

(A) ProA elution profile from conditioned medium expressing anti-pTau C21-ABS (B) SDS-PAGE analysis of anti-pTau C21-ABS ProA elution fractions indicating the location of heavy and light chains (C) SEC elution profile of ProA-enriched anti-pTau C21-ABS (D) Loading series of final purified anti-pTau C21 ABS IgG with heavy and light chains indicated

**Figure S2. Anti-pTau C21-ABS Fab generation and crystallisation**

(A) SDS-PAGE analysis showing anti-pTau C21 Fab generation time-course (lanes 3-8) from purified IgG (lane 2) (B) Crystals of anti-pTau C21-ABS Fab in complex with pTau peptide grew in 29 days.

**Figure S3 Comparison of pTau peptide positioning over anti-pTau CDR loops**

Cartoon representation of the anti-pTau WT and C21-ABS paratope/epitope interface. Fabs are displayed in grey, LCDR1 in light blue, LCDR2 in blue and LCDR3 in purple. HCDR1 is displayed in brown, HCDR2 in red and HCDR3 in pink. The pTau peptide is displayed in stick configuration.

**Figure S4 Amino acids contributing to electrostatic surface charge in anti-pTau C21-ABS**

Anti-pTau C21-ABS-C21 Fab is displayed as a surface space-fill plot with electrostatic surface-charge potential at 60% transparency. Positive surface charge is displayed in blue and negative surface charge is displayed in red. The three amino acids R53, D100 and E100F contributing to key surface-charge areas, as well as Y32, are displayed in stick with labels indicating position. The pTau peptide is displayed in green in stick configuration.

**Table S1 Table of Refinement Statistics**

| Data collection | |
| --- | --- |
| Space group | P2_1_ |
| Unit cell dimensions  a, b, c (Å)  α, β, γ (°) | 52.22, 76.33, 119.05  90, 90.36, 90 |
| Wavelength (Å) | 1.03 |
| Resolution (Å) | 76.6 – 2.6 (2.74 - 2.6) |
| Number of unique reflections | 29,183 (4,234) |
| 〈I/σ〉 | 9.5 (2.3) |
| Completeness (%) | 99.7 (99.7) |
| Redundancy | 3.4 (3.4) |
| R_merge_^a^ | 0.079 (0.41) |
| Refinement | |
| Resolution (Å) | 64.47 – 2.6 (2.69 - 2.6) |
| R_cryst_/R_free_ (%)^b^ | 21.1/23.1 (25.2/28.0) |
| Number of reflections  Working set  Test set | 27,798  1,368 |
| rmsd from ideal values  Bond lengths (Å)  Bond angles (°) | 0.009  1.165 |
| Number of atoms  Protein  Solvent | 6,417  329 |
| Mean B values (Å^2^) | 56.88 |
| Ramachandran plot^c^  Preferred regions (%)  Allowed regions (%)  Disallowed (%) | 98.02  1.63  0.35 |

Numbers in parentheses are for the highest-resolution shell.

^a^ Rmerge=Σ|I−〈I〉|/ΣI, where I is the intensity of the

measured reflection, and 〈I〉 is the mean intensity of all

measurements of this reflection.

^b^ Rcryst=Σ‖F_obs_|−|F_calc_‖/Σ|F_obs_|, where Fobs and F_calc_ are

the observed and calculated structure factors, respectively. R_free_ is calculated for 5% of reflections randomly chosen prior to

refinement.

^c^ The Ramachandran plot was calculated with Coot.

**Table S2 Alignment of anti-pTau WT and C21-ABS CDR regions**

|  | CDRL1 | CDRL2 | CDRL3 | CDRH1 | CDRH2 | CDRH3 |
| --- | --- | --- | --- | --- | --- | --- |
| Human Germline | QGDRSYYA | GKNNRPS | NSRDSSGNHVV | SSYAMS | AAISGSGGSTYYADSVKG |  |
| WT | SGSDYDYG | WNDKRPS | GAYDGSAGGGI | SSYQMM | SGITSRGGVTGYGSAVKG | PALDSDQCGFPEAGCIDA |
| C21-ABS | QGDDSYYG | GNDNRPS | GAYDSSGGGGI | SSYQMM | AGITGRGGVTGYADSVKG | PALDSDQCGFPEAGCIDA |

Table showing humanisation of anti-pTau C21-ABS. Germline residues are displayed in black and chicken residues are displayed underlined in black.
