# Supplementary figures and images for: Crystal structure of ultra-humanized anti-pTau Fab reveals how germline substitutions humanize CDRs without loss of binding’
Source: Sci Rep. 2022 May 24;12:8699. doi: 10.1038/s41598-022-12838-6 (PMC9130293; doi:10.1038/s41598-022-12838-6)

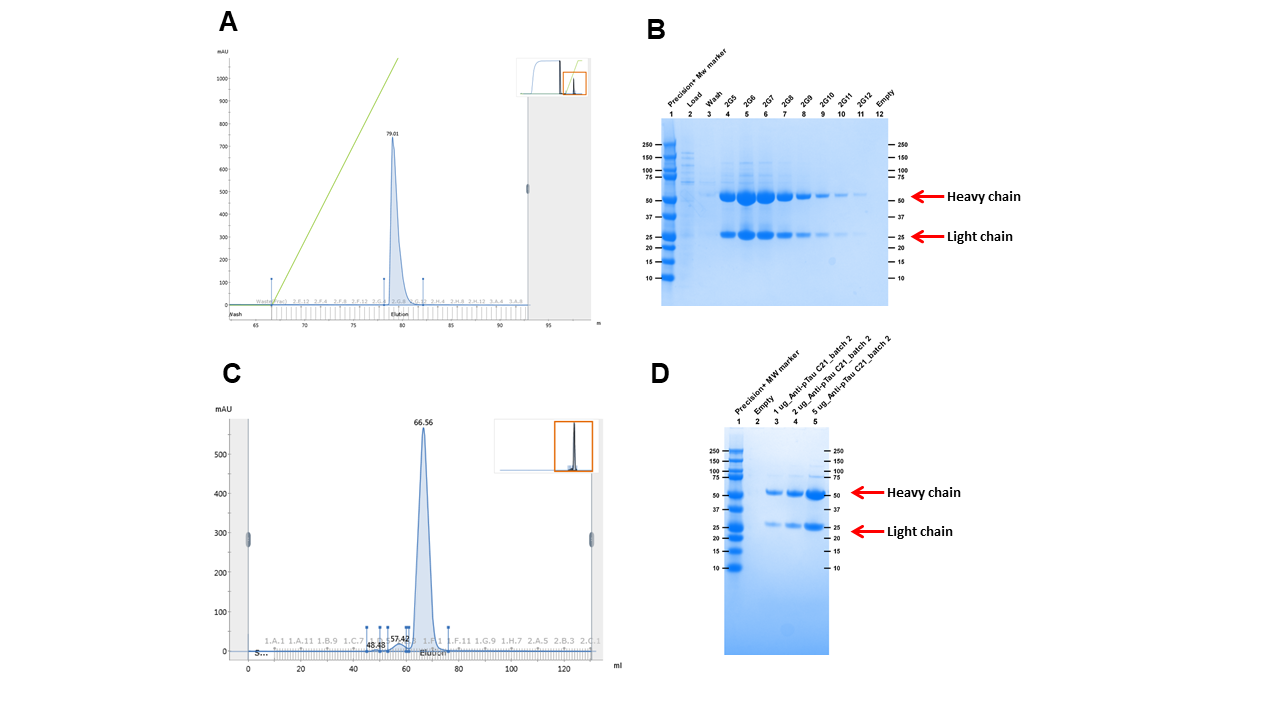

Supplement: Supplementary file 2 — Supplementary Information 2. [file 41598_2022_12838_MOESM2_ESM.tif]

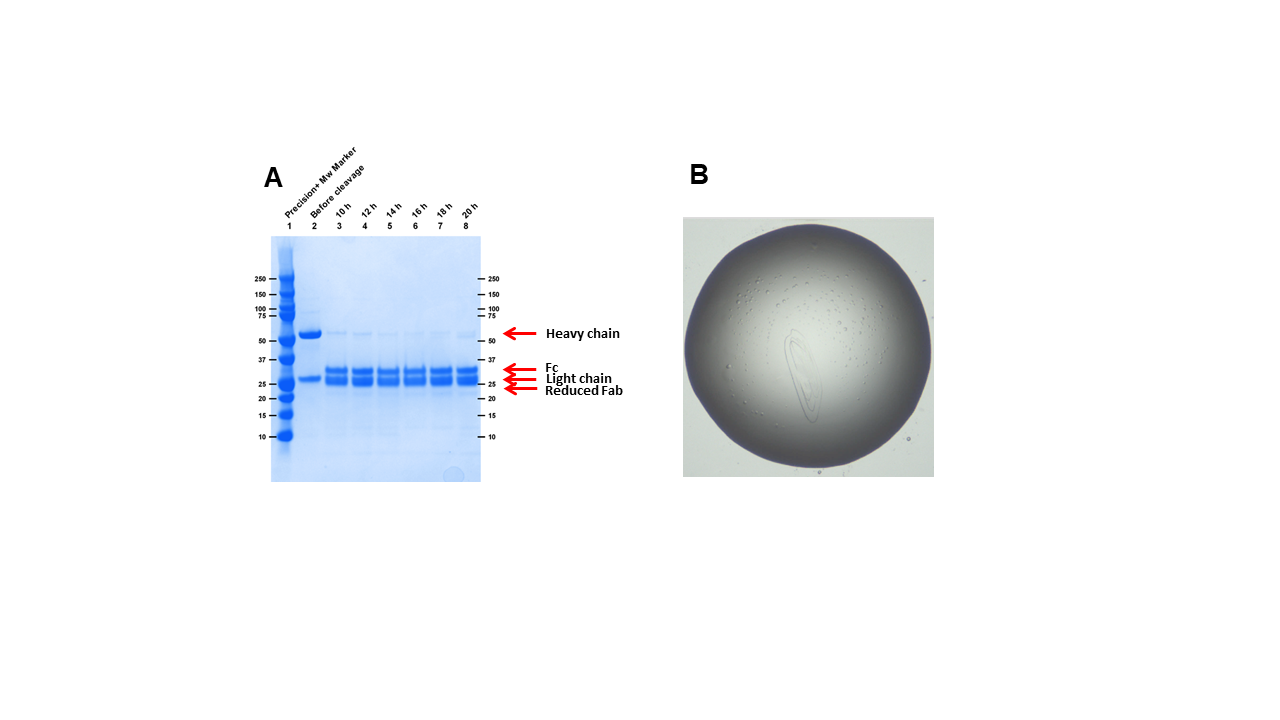

Supplement: Supplementary file 3 — Supplementary Information 3. [file 41598_2022_12838_MOESM3_ESM.tif]

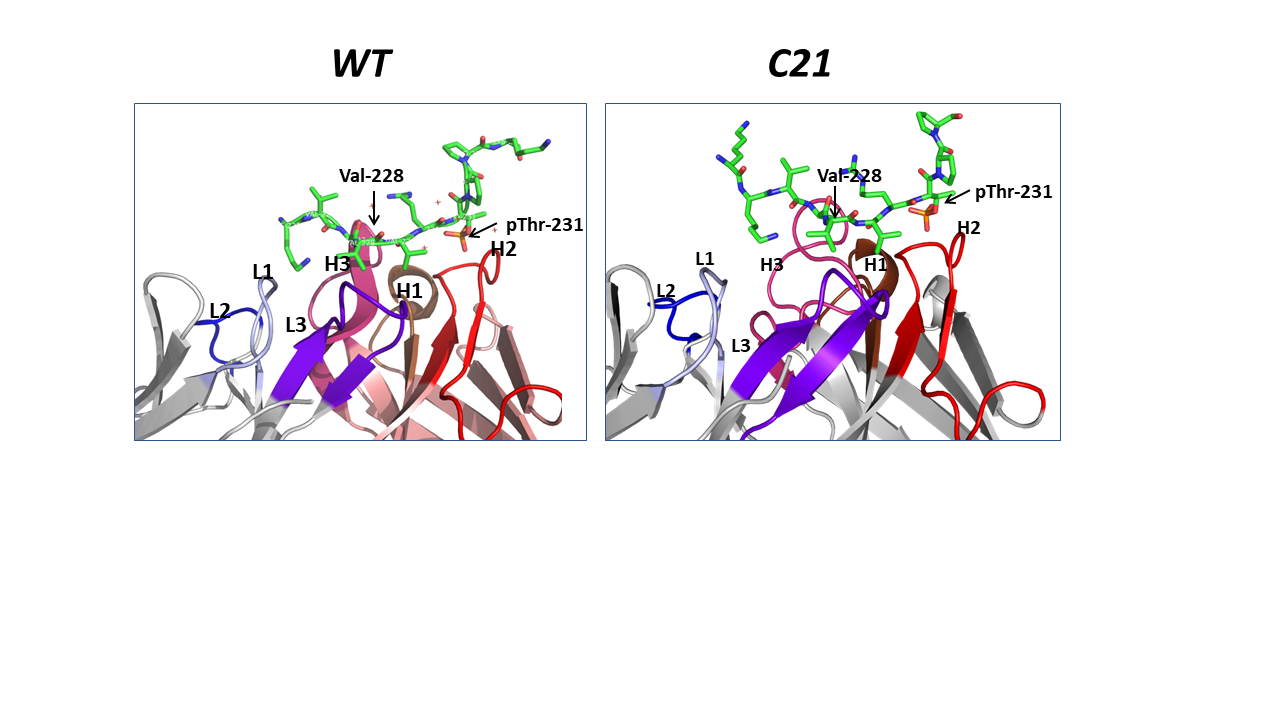

Supplement: Supplementary file 4 — Supplementary Information 4. [file 41598_2022_12838_MOESM4_ESM.tif]

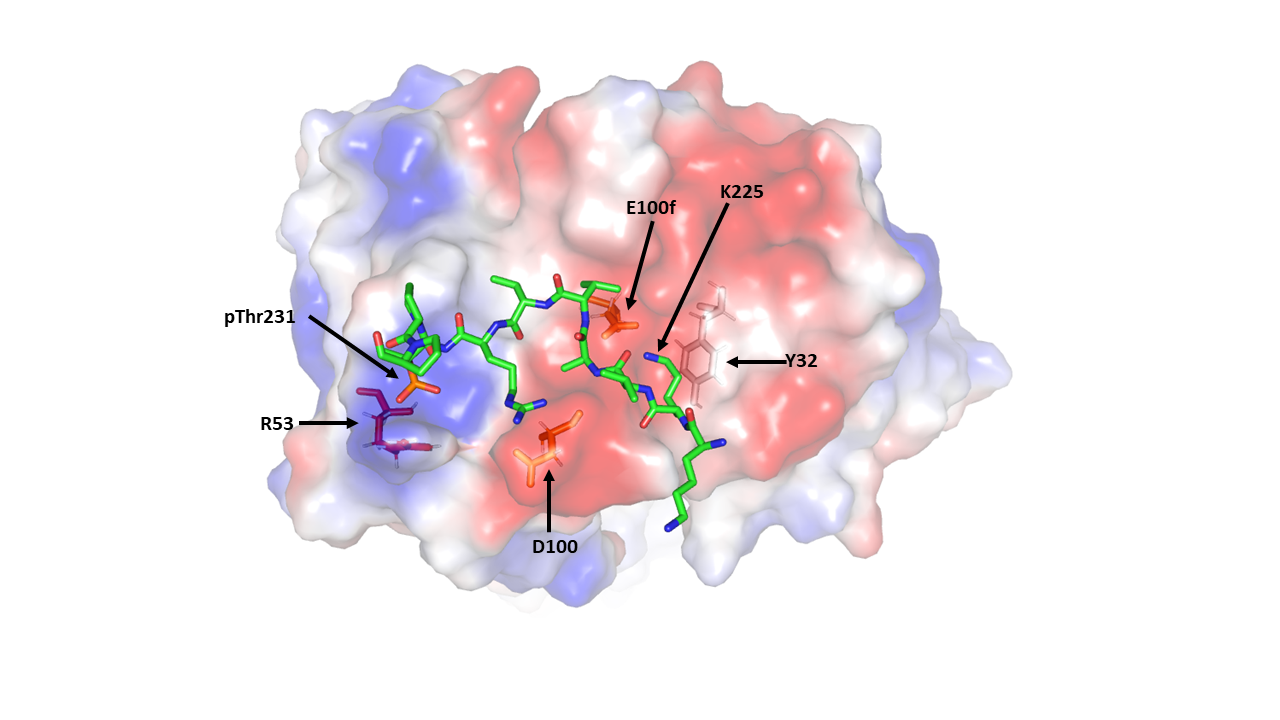

Supplement: Supplementary file 5 — Supplementary Information 5. [file 41598_2022_12838_MOESM5_ESM.tif]
